# Passively transferred human NMO-IgG exacerbates demyelination in mouse experimental autoimmune encephalomyelitis

**DOI:** 10.1186/1471-2377-13-104

**Published:** 2013-08-08

**Authors:** Harleen Saini, Robert Rifkin, Michael Gorelik, Hwa Huang, Zachary Ferguson, Melina V Jones, Michael Levy

**Affiliations:** 1 Department of Neurology, Johns Hopkins University, Baltimore, MD 21287, USA; 2 Neurology, Johns Hopkins University, 600 N. Wolfe Street, Pathology 509, Baltimore, MD 21287, USA

**Keywords:** Neuromyelitis optica, Aquaporin-4, NMO-IgG, Astrocytes, Experimental autoimmune encephalomyelitis

## Abstract

**Background:**

Neuromyelitis optica (NMO) is a devastating inflammatory disorder of the optic nerves and spinal cord characterized by frequently recurring exacerbations of humoral inflammation. NMO is associated with the highly specific NMO-IgG biomarker, an antibody that binds the aquaporin-4 water channel. Aquaporin-4 is present on glial endfeet in the central nervous system (CNS). In humans, the NMO-IgG portends more frequent exacerbations and a worse long-term clinical outcome.

**Methods:**

We tested the longer-term outcome of mice with EAE injected with NMO-IgG and followed them for 60 days. Clinical exams and pathology of the spinal cord and optic nerves were compared to mice that received control human IgG.

**Results:**

Passively transferred human NMO-IgG leads to more severe neurology disability over two months after onset of disease. Clinical worsening is associated with an increased concentration of large demyelinating lesions primarily to subpial AQP4-rich regions of the spinal cord.

**Conclusions:**

NMO-IgG is pathogenic in the context of EAE in mice.

## Background

Neuromyelitis optica (NMO) is a devastating neuroinflammatory disorder that preferentially targets the optic nerves, brainstem and spinal cord [1]. Also known as Devic’s disease, NMO is associated with the highly specific NMO-IgG antibody found in up to 63% of patients [2]. The target of the NMO-IgG is the aquaporin-4 (AQP4) water channel expressed in multiple tissues in the body. AQP4 is the major aquaporin found in the CNS and is highly localized to the endfeet of astrocytes, especially along the pia limitans and on the abluminal surface of blood vessels in the brain [3].

The NMO-IgG is hypothesized to be pathogenic; binding of the antibody to its glial target triggers a humoral inflammatory cascade involving IgG, IgM, complement deposition and recruitment of neutrophils and eosinophils [4]. This model of disease is supported by two reports of passively transferred NMO-IgG in which the NMO-IgG exacerbates behavioral signs of rat experimental autoimmune encephalomyelitis (EAE) and induces a pathology similar to human NMO: areas of acute inflammation with granulocytes, a dramatic loss of aquaporin-4 staining and complement deposition [5,6]. While EAE induced by myelin basic protein in complete Freund’s adjuvant (CFA) in Lewis rats generally leads to a complete neurologic recovery [7], EAE induced by myelin oligodendrocyte glycoprotein peptide 35–55 (MOG_35-55_) in C57Bl6 mice causes demyelination and axon loss in the spinal cord with limited behavioral recovery, the latter of which may better represent a more suitable animal model system for severe human neuromyelitis optica disease.

One of the hallmarks of the NMO-IgG seropositive testing in humans with NMO disease is the prognostic implication of more frequent recurrences and worse neurologic outcome with increased disability compared to individuals with NMO whose serum does not react with AQP4 [8]. In our study, we tested the consequences of NMO-IgG in mouse EAE passively transferred at disease onset on long-term outcome and found that the NMO-IgG results in a worse neurological outcome which is maintained as late as two months after immunization. Pathological evaluation revealed larger, primarily subpial demyelinated lesions in the spinal cord and optic nerves of EAE mice receiving passively transferred NMO-IgG.

## Methods

### Animals

Adult female C57/BL6 mice between 6 – 8 weeks of age were purchased from The Jackson Laboratory and housed in a 12-hour artificial light–dark cycle and had ad libitum access to food and water. The Johns Hopkins Institutional Animal Care and Use Committee approved all experimental procedures.

### Samples

Human IgG fractions were purified from the plasma of patients undergoing plasma exchange using a resin based purification method (Melon Gel IgG Purification kit, Thermo Scientific) two days prior to injection. The purified IgG was concentrated by spin column centrifugation (Amicon Ultra, 100kD MW cut off) and the final protein concentration was adjusted to 25 mg/ml for 100 μl intra-peritoneal injection. All NMO patients tested seropositive for the NMO-IgG by the Mayo clinical NMO-IgG assay and the NMO plasma samples from 3 patients were pooled prior to purification. Human control IgG fraction (control-IgG) was obtained from a non-NMO patient undergoing plasma exchange for ABO incompatibility. All samples were obtained through a protocol approved by the Johns Hopkins Institutional Review Board (NA_ 00003551) in a de-identified manner with informed consent to use the samples for research.

### EAE induction and scoring

Experimental autoimmune encephalomyelitis (EAE) was induced in 3 groups of 10 mice. Each animal received a single subcutaneous injection of one hundred microliters of an emulsified solution of 1:1, 5 mg/ml Myelin Oligodendrocyte Glycoprotein (MOG) peptide 35–55 in phosphate buffered saline (PBS) and incomplete Freund’s adjuvant containing 12.5 mg/ml heat-killed *Mycobacterium tuberculosis*; each animal therefore received 250 μg MOG_35-55_ and 625 μg *M. tuberculosis* (day 0). Pertussis Toxin (300 ng) was administered intraperitoneally on days 0 and 2. Animals were weighed daily and scored on a standardized 5-point EAE disability scale by a blinded examiner [9]. A series of 4 intraperitoneal injections of human IgG purified from either pooled NMO plasma or control human plasma were administered on days 13, 14, 18, and 19 for a total of 10 mg/animal [5], with the initial pair of injections corresponding to onset of EAE disease and the second pair of injections provided during the initial remission of EAE. Vehicle controls received an equal volume of PBS.

### Tissue processing and histology

Animals were anesthetized with isofluorane and perfused via cardiac puncture first with PBS and then with freshly prepared 4% formaldehyde solution. The optic nerves and spinal cords were harvested, fixed overnight, cryopreserved in 30% sucrose and frozen for sectioning. After embedding tissue in in O.C.T. Compound (Tissue-Tek^®^ ), ten to thirty micron slices sections were mounted on Superfrost Plus Microscope Slides (Fisher brand). The first cohort of animals was sacrificed 20 minutes after the last intraperitoneal injection of human IgG for the purpose of tracking human antibody entrance into the mouse central nervous system. The second cohort of animals was sacrificed and their tissue was prepared in a similar fashion on day 62 post-EAE induction.

### Eriochrome cyanine staining for myelin

Eriochrome cyanine was used to identify demyelinating lesions in the sectioned tissue. Slides with frozen sections were thawed at room temperature. Eriochrome cyanine solution was prepared by dissolving eriochrome cyanine in 450 ml 0.5% H_2_SO_4_(0.2%) and 10% FeCl_3_ added to a final concentration of 0.4%. The sectioned tissue was hydrated by serial washes in 100% ethanol, 95% ethanol, 70% ethanol and distilled water for 10 minutes each and then immersed for 15 minutes in eriochrome cyanine solution. After staining, differentiation was carried out in freshly made 0.1% NH_4_OH for 20–30 seconds and halted by thorough washing in distilled water. Slides were mounted as described in the preceding section.

### Immunohistochemistical staining

Immunohistochemical staining was performed by immersing 20–30 μm sections of mouse CNS tissue in blocking solution for 2 hours at room temperature as indicated in Table 1 before incubation with primary antibody overnight at 4°C. Sections were then washed three times in PBS prior to incubation with a secondary antibody conjugated with either Alexa Fluor 488 or 555 (Invitrogen) at room temperature for 1 hour. Slides were then washed and mounted with fluorescence mounting media (Dako) or Fluoro-gel (Electron Microscopy Sciences).

**Table 1:** Details of the primary antibody reagents used for immunohistochemical experiments in these studies

**Primary antibody**	**Antigenic target**	**Concentration**	**Blocking solution**	**Manufacturer**	**Catalog number**
Anti-Aquaporin 4, C-terminus	Aquaporin-4	1:200	5% BSA-PBS + 1:100 NGS + 0.1% Triton X-100	Millipore	AB3594-200UL
Purified Rat anti-mouse CD45	All cells of hematopoietic origin, except erythrocytes	1:50	5% BSA-PBS + 1:100 NGS	BD Pharmingen	550539
Purified Rat anti-mouse CD5	Thymocytes, T lymphocytes, thymic NK-T cells, subset of B lymphocytes	1:50	5% BSA-PBS + 1:100 NGS + 0.1% Triton X-100	BD Pharmingen	553017
Purified Rat anti-mouse CD45R (B220)	B lymphocytes	1:50	5% BSA-PBS + 1:100 NGS + 0.1% Triton X-100	BD Pharmingen	550286
Purified Rat anti-mouse Ly-6G and Ly-6C	Granulocytes	1:50	5% BSA-PBS + 1:100 NGS + 0.1% Triton X-100	BD Pharmingen	550291
Purified Goat anti-human	Human IgG	1:250	5% BSA-PBS + 1:100 NGS	Invitrogen	A-11013

### Myelin quantification

Six to eight areas of eriochrome-stained spinal cord were photographed at high resolution (4080x3072) using a 4x objective and background corrected to correct for uneven illumination. Image analysis was performed in a blinded fashion. Using ImageProPlus5 software, two measurements were acquired from each image: (1) a total measure of the white matter area (including eriochrome-stained regions and demyelinated areas) and (2) a measure of each demyelinated lesion’s area, outlined as separate areas of interest (AOIs). During analysis, each lesion was noted to be largely bordered by the pia mater or not; while most EAE lesions contact the pia, this analysis was to indicate lesions whose major axis contacted the pia. For total demyelinated area, lesioned areas were summed. For percent demyelinated area, this measure was divided by the total white matter area. “Large pial lesions” were classified as those comprised of ≥10,000μm^2^. For percent large pial lesions, the total area of these large lesions was divided by the total demyelinated area for that animal to demonstrate the prevalence of these large subpial confluent lesions in these mice. For percent pial lesions, the area of all lesions blindly classified as primarily pial was summed and divided by the total demyelinated area for that animal.

### Statistics

For comparison of EAE scores, a non-parametric (Mann–Whitney U-test) was performed. For comparison of demyelinated areas, Student t-tests were performed. Data was analyzed and depicted using GraphPad 5.0 software. Results were deemed statistically significant for p<0.05.

## Results

**Figure 1:**
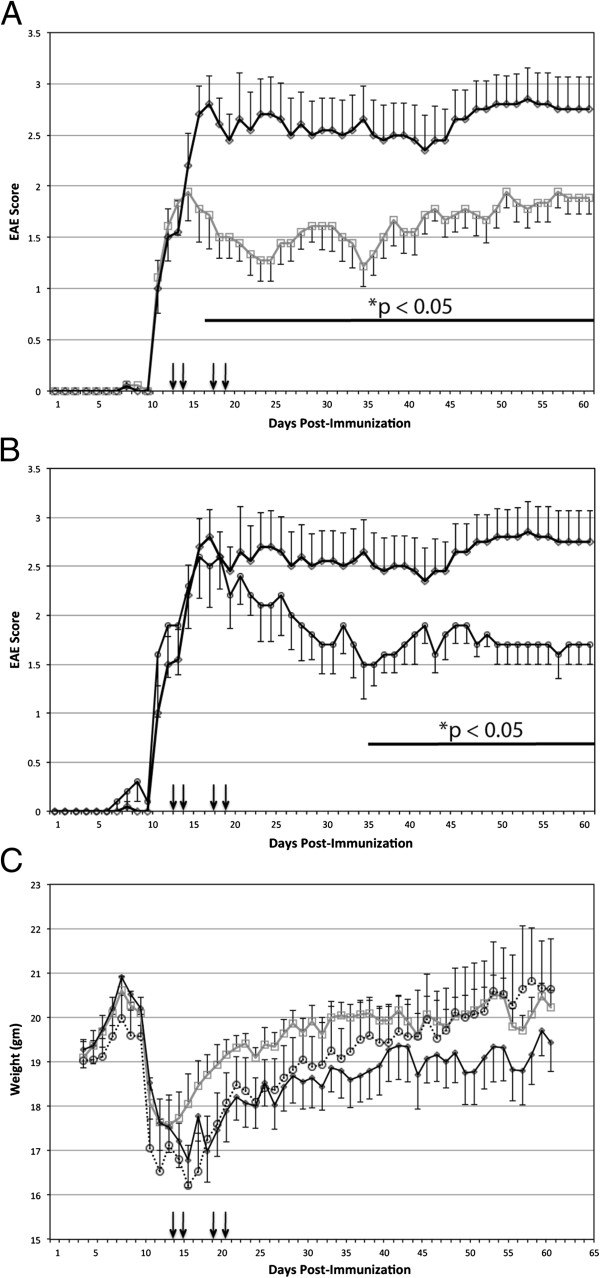
**Experimental autoimmune encephalomyelitis (EAE) with passive transfer of the NMO-IgG, control IgG and vehicle saline control. (A)** EAE scores comparing mice receiving NMO-IgG (diamonds, black line) versus vehicle saline control (squares, grey line) with 4 injections on days 13, 14, 18, and 19 as indicated by the black arrows. EAE scores were statistically different starting from day 15 post-immunization. **(B)** EAE scores comparing mice receiving the NMO-IgG (diamonds, black line) versus control IgG (circles, black line). EAE scores were statistically different starting from day 25 post-immunization. **(C)** Weight of NMO-IgG mice (diamonds, black line), control IgG mice (circles, dashed line) and vehicle saline control mice (squares, grey line) during course of EAE. Sample size = 10 mice in the NMO group, 9 in the vehicle contorl group, 5 in the control IgG group. Experiment repeated with similar results.

Compared to vehicle controls, EAE mice receiving passively transferred NMO-IgG developed significantly worse EAE scores within 24 hours of injection and remained stably worse for 2 months (Figure 1A). EAE mice passively transferred with control-IgG similarly worsened in the first 24 hours but recovered to EAE scores similar to vehicle-treated controls over the next two months (Figure 1B). Exacerbated weight loss persisted in the NMO-IgG-treated mice during the 2-month experiment, thus reflecting the worsening EAE scores during that time period (Figure 1C).

**Figure 2:**
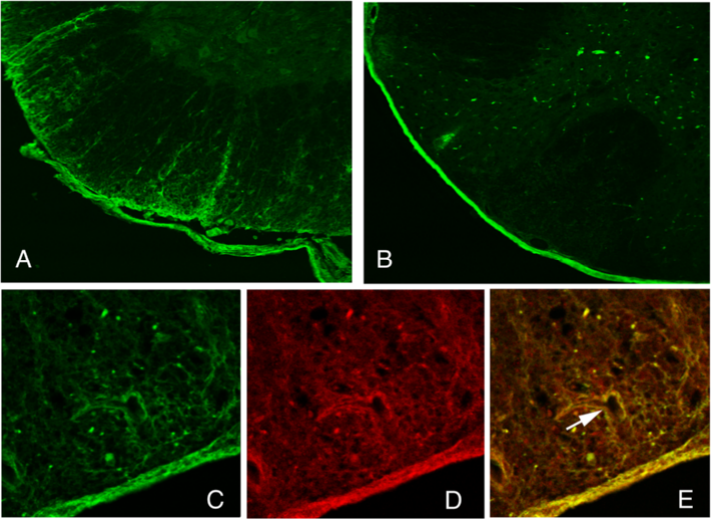
**Spinal cord sections from mouse 24 hours after 4 injections of NMO-IgG on days 13, 14, 18, and 19 (A) versus control IgG (B) showing NMO-IgG penetration into the parenchyma of the spinal cord as shown by anti-human IgG staining (green).** Sections were costained with anti-human IgG **(green, C)**, anti-AQP4 antibody **(red, D)** showing co-localization in areas of AQP4 expression especially around blood vessels **(arrow in merged image, E)**.

To determine the target(s) of human IgG injected into EAE mice, we sacrificed a cohort of mice 24 hours after the final of four intraperitoneal injections and stained tissue sections for anti-human antibody. In addition to positive staining in non-CNS tissue such as the lung and kidney, we found significant levels of anti-human antibody staining in the spinal cord and optic nerve, which are the predominant sites of NMO disease in humans (Figure 2A). The control-IgG was also found in the CNS, but largely in the meninges and not within the parenchyma of the spinal cord (Figure 2B). Co-staining with anti-AQP4 revealed colocalization with anti-human antibody, especially around blood vessels and in the pia-glia limitans of the spinal cord (Figures 2C-E). This suggests that human NMO-IgG antibodies injected intraperitoneally can reach their target in the spinal cord of inflamed mouse spinal cord in EAE. Forty-three days following the last intraperitoneal injection of NMO or control-IgG, staining for human IgG in the spinal cords was indistinguishable from vehicle control mice (injected with PBS), confirming that the human antibodies were removed from circulation.

**Figure 3:**
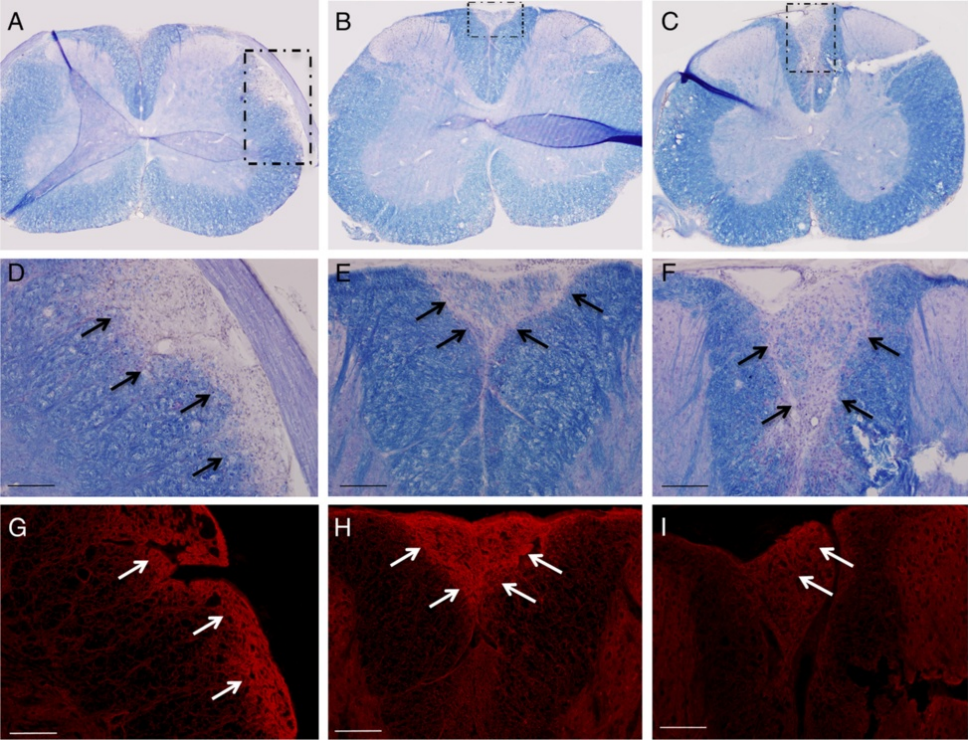
**Sections from spinal cords of mice 62 days after injection of NMO-IgG (A, D, G), human IgG controls (B, E, H) and vehicle saline controls (C, F, I). Eriochrome staining shows large areas of pale blue demyelination in the NMO-IgG group (A, magnified in D) as indicated by the arrows.** Typical EAE areas of posterior column demyelination were present in the control-IgG **(B, magnified in E)** and vehicle control **(C, magnified in F)** groups. Aquaporin-4 staining (red) in all groups were similar and appeared increased in areas of demyelination. Scale bars in D-I represent 100 μm.

Sixty-two days after induction of EAE, 43 days following the last IgG infusion, the group that received the NMO-IgG had numerous large subpial demyelinated lesions that were located in the lateral and ventral columns of the spinal cord (Figure 3). Vehicle and control-IgG treated animals had typical demyelinated lesions which extended into the deeper white matter areas, in contrast to NMO-IgG lesions that seemed to be bordered primarily by the pia. Aquaporin-4 staining of these sections revealed homogenous staining but with an increase in AQP4-immunoreactivity in areas of demyelination in all three groups (Figure 3).

**Figure 4:**
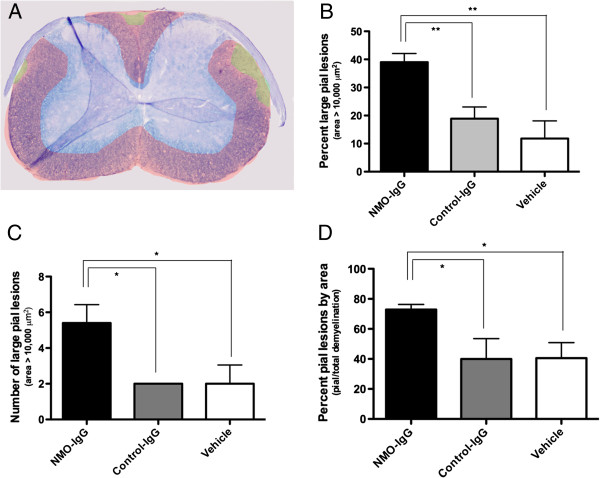
**Quantification and characterization of demyelinated lesions in the spinal cord were based on manual outlines of the total myelinated area (blue) and the demyelinated area (red/yellow) as shown in A.** The number of pial lesions greater than 10,000 μm^2^ made up nearly 40% of all the demyelinated lesions in the NMO-IgG group **(B)**. In addition, the total number of pial lesions in each NMO-IgG mouse on average was higher **(C)**. Accounting for pial lesions of all sizes, the NMO-IgG group had almost double the number compared to the control-IgG and vehicle groups **(D)**.

The overall proportion of demyelinated area was not significantly different between the 3 groups (NMO-IgG vs. CTL-IgG p=0.43, vs. Vehicle p=0.70). However, there were a significantly greater number of large lesions (defined as ≥10,000 μm^2^) that were primarily subpial in the NMO group (5.4± 1.2) compared to the control-IgG (2.0± 0.0) or the vehicle control group (2.0± 1.2) (p=0.048 and 0.049 respectively; Figure 4C). When expressed as a percentage of the total demyelinated area, large subpial lesions comprised a greater proportion of the lesioned area in the NMO-IgG group (39.0 ± 3.5%) than in the control-IgG (18.9 ± 5.1%) or the vehicle-treated group (11.8 ± 7.0%) (p<0.008 and p<0.005 respectively) (Figure 4). Considering pial lesions of all sizes, 72.9 ± 3.8% of all demyelinated area in the NMO-IgG group was subpial compared to 39.9 ±16.7% in the control-IgG group (p=0.02 vs. NMO-IgG) and 40.6 ± 11.5% in the vehicle control group (p=0.02 vs. NMO-IgG). Simply injecting non-specific IgG into mice did not alter lesion localization, since the pattern of demyelination in the control-IgG and vehicle control groups were not significantly different from each other (p=0.97).

**Figure 5:**
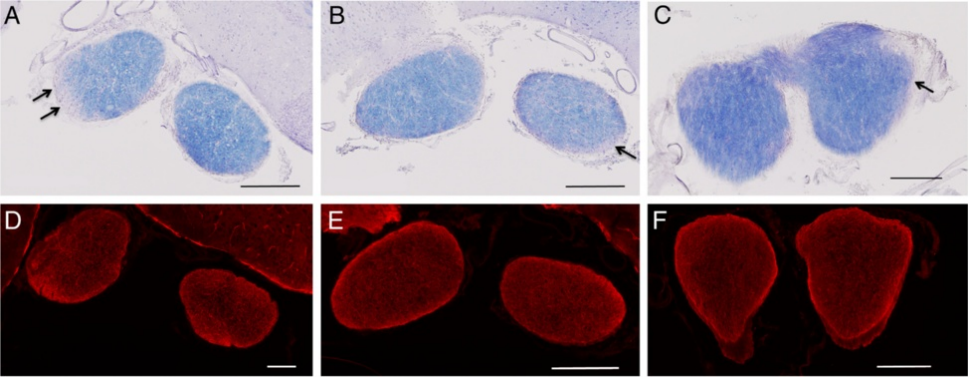
**Optic nerve sections were stained for myelin by eriochrome (blue stain) and AQP4 expression 62 days after injection of human IgG or saline.** The mice that received injections of NMO-IgG show areas of pale blue demyelination **(arrows in A)**. The control-IgG group had some demyelination in the optic nerves **(arrows in B)** and vehicle controls had only few visible demyelinating lesions **(panel C)**. Similar to the spinal cord, AQP4 staining (anti-AQP4, Santa Cruz, white) was increased in all groups in areas of demyelination **(sections D-F)**. Scale bars represent 100 μm.

Similar to the spinal cord, optic nerves from the NMO-IgG-treated group showed subpial demyelinating lesions (Figure 5). The control-IgG and vehicle control groups also had occasional areas of demyelination in the optic nerve and quantitative comparison proved difficult with this small amount of optic nerve tissue. As in the spinal cord, AQP4 staining of optic nerve sections revealed normal homogenous staining of all sections with an increase in staining in areas of demyelination in all three groups (Figure 5).

**Figure 6:**
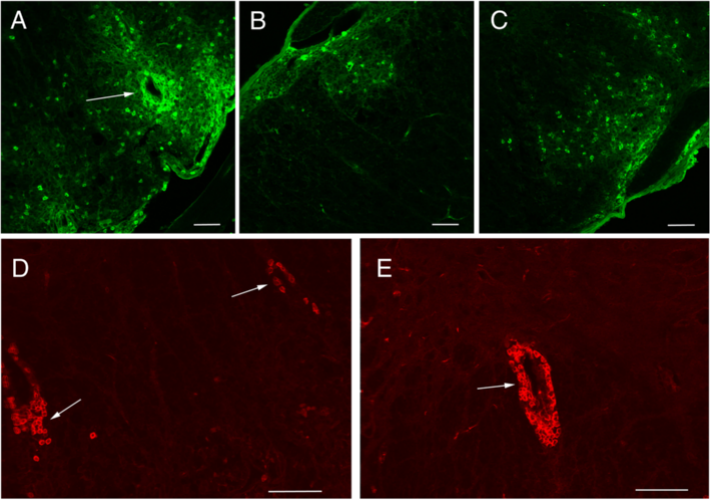
**Staining for the types of immune cells in acute lesions (24 hours after last human IgG injection) identified heavy infiltration by granulocytes (anti-Ly6G, BD Pharmingen, green) in parenchymal spinal cord lesions of mice that received NMO-IgG (A) especially around blood vessels as indicated by the arrow, compared to occasional scattered granulocytes in control IgG (B) and saline vehicle controls (C).** T-cell infiltration was similar among all groups. The arrows identify T-cells (anti-CD5, BD Pharmingen, red) in lesions of the NMO-IgG mice **(D)** and isotype control IgG **(E)**. Scale bars represent 100 μm.

In most models of rodent EAE, the majority of white blood cells in CNS lesions are T-cells, while human NMO pathology is characterized by the presence of granulocytes, including neutrophils and eosinophils in demyelinating lesions, along with T cells, a few B-cells, and macrophages. We found that several early (20 days post-immunization) lesions in the NMO-IgG-treated group developed lesions that were infiltrated by granulocytes in addition to typical EAE lesions identified by T cells (Figure 6). The spinal cords of control-IgG treated mice showed sporadic granulocytes, although not in high numbers or in a perivascular distribution. While further study is needed to quantify the granulocyte infiltration, it appears that granulocytes within EAE lesions may have contributed to the worsening of both the NMO and control-IgG groups. At 62 days after immunization, rare inflammatory EAE lesions in all three groups were still evident with typical infiltration by T-cells, but granulocytes were no longer present.

## Discussion

Our mouse model of NMO resembles human NMO in several ways. First, the neurological phenotype of mouse EAE worsens in the presence of NMO-IgG and remains persistently worse for the duration of our 2-month study. This correlates with large subpial demyelinated lesions in the spinal cord. Second, the optic nerves and spinal cord are the predominant sites of pathology, similar to human NMO disease. Thirdly, pathologic evaluation of EAE mice exposed to NMO-IgG showed evidence of infiltration by granulocytes in acute lesions.

In the context of ameliorating NMO severity, one may conclude from this study that NMO-IgG may be harmful when present in acute inflammatory lesions of the CNS and should be targeted for removal. Data on patients is mixed with regards to the correlation of NMO-IgG titer with either the severity of an acute attack or the degree of recovery following treatment [10,11]. However, there is good evidence that presence of the NMO-IgG is predictive of more frequent relapses and a worse neurologic outcome [8], the latter of which we observed in our mouse model.

There has been debate on the role of the NMO-IgG as a pathogenic antibody versus a biomarker of disease [12-14]. In our model, there is strong evidence in favor of the NMO-IgG as a contributor to the neurological pathology and behavioral outcome. As in rat models of NMO [5,6], there is no effect of the NMO-IgG in the absence of EAE in this strain of mouse and there is no penetration of NMO-IgG into the CNS parenchyma in the absence of EAE. However, NMO-IgG appears to convert the pathology and disease course in mice from acute behavioral disease with small lesions within superficial white matter of the dorsal columns to confluent pial lesions in the dorsal and lateral spinal cord. Although the total demyelination is similar, large confluent lesions involving the dorsolateral corticospinal tracts may lead to greater disability in this mouse model. We also found limited evidence for perivascular granulocyte recruitment in the NMO-IgG treated animals but the role of these cells in altering the distribution of lesions and in causing persistent behavioral disease in our EAE mice needs further study. Our hypothesis is that NMO-IgG targets AQP4, which is preferentially expressed on the astrocytic foot processes of the pia-glia limitans. As a result of the antibody binding, a humoral-mediated inflammatory process ensues that includes granulocyte infiltration. It is temporary because the human NMO-IgG is cleared shortly after passive transfer. Interestingly, while behavioral disease persisted for 2 months, human IgG and granulocytes were not detectable in the CNS at the 2-month time point. This suggests that transient exposure to the NMO-IgG has long-lasting consequences. This might explain why NMO-IgG titer is not easily linked to disease severity: transient spikes in titer coinciding with blood–brain permeability in the spinal cord and optic nerve may cause tissue damage and disease, but the overlap of these two events may be rare.

In contrast to our mouse model, rats with EAE that received control-IgG did not manifest behavioral worsening defined as a higher EAE disability score [5,6]. In addition, some patients’ NMO-IgG have more toxic potential than others [15]. While this may be due to the presence of autoantibodies, which are not reactive to the animal model species being used, it is still unclear as to why we observed transient worsening of behavioral signs in EAE mice injected with the control-IgG [16]. The patient we isolated our control-IgG from suffered from chronic kidney disease that required plasma exchange to remove ABO incompatible antibodies from circulation prior to kidney transplant. In addition to not being positive for AQP4-immunoreactivity, we found no evidence of other autoantibodies binding to antigens in the mouse CNS. The observed transient worsening in mice receiving the control-IgG did not significantly alter the distribution of lesions as did the NMO-IgG. Because this worsening was not observed in rat models of EAE, this effect of control-IgG could be unique to this patient’s IgG. The difference in rodent species could also influence the course of EAE and reactivity of autoantibodies.

Another difference between this mouse NMO model and the previous rat NMO models is that the behavioral worsening in our mouse model was not accompanied by AQP4 depletion. This could mean that higher EAE disability score with NMO-IgG does not require overt depletion of astrocytes or of AQP4 from lesions but does not rule out AQP4 involvement. Interestingly, even severe depletion of AQP4 and astrocytes from lesioned areas in rat animal models does not lead to myelin damage, suggesting that myelin can survive at least transient loss of AQP4. The long term consequences of short term NMO-IgG exposure in rat models has not yet been reported. Also, purified preparations of NMO-IgG have been difficult to obtain due to the polyclonal nature of the antibody and the uncertainty about which antigenic targets on AQP4 are important in the pathogenesis of disease [17]. Further work in this area will allow better specificity of passive transfer studies in NMO.

## Conclusions

In this experimental model of EAE in C57/BL6 mice in which passively transferred IgG from NMO patients is infused at onset of neurologic disease, we conclude that the anti-AQP4 antibody contributes to the pathogenicity by targeting subpial astrocytes leading to large, confluent demyelinated lesions in the spinal cord and optic nerve.

## Competing interest

All authors declare that they have no competing interests.

## Authors’ contribution

HS led the animal and histology experiments, analyzed the data and contributed to the writing and editing of the manuscript. RR participated in the histology preparation and analysis, and edited the manuscript. MG participated in the animal experimentation and edited the manuscript. HH participated in the histology preparation and analysis and edited the manuscript. ZF participated in the histology preparation and analysis and edited the manuscript. MVJ participated in the animal experimentation, histology and lesion quantification, as well as editing the manuscript. ML oversaw the project, designed the experiments, analyzed the data and wrote the manuscript. All authors read and approved the final manuscript.

## Pre-publication history

The pre-publication history for this paper can be accessed here:

http://www.biomedcentral.com/1471-2377/13/104/prepub

## References

[B1] Graber DJ Levy M Kerr D Wade WF Neuromyelitis optica pathogenesis and aquaporin 4 J Neuroinflammation 2008 5 22 10.1186/1742-2094-5-22 18510734 PMC2427020

[B2] McKeon A Fryer JP Apiwattanakul M Lennon VA Hinson SR Kryzer TJ Lucchinetti CF Weinshenker BG Wingerchuk DM Shuster EA Pittock SJ Diagnosis of neuromyelitis spectrum disorders: comparative sensitivities and specificities of immunohistochemical and immunoprecipitation assays Arch Neurol 2009 66 1134 1138 10.1001/archneurol.2009.178 19752303

[B3] Roemer SF Parisi JE Lennon VA Benarroch EE Lassmann H Bruck W Mandler RN Weinshenker BG Pittock SJ Wingerchuk DM Lucchinetti CF Pattern-specific loss of aquaporin-4 immunoreactivity distinguishes neuromyelitis optica from multiple sclerosis Brain 2007 130 1194 1205 10.1093/brain/awl371 17282996

[B4] Lucchinetti CF Mandler RN McGavern D Bruck W Gleich G Ransohoff RM Trebst C Weinshenker B Wingerchuk D Parisi JE Lassmann H A role for humoral mechanisms in the pathogenesis of Devic's neuromyelitis optica Brain 2002 125 1450 1461 10.1093/brain/awf151 12076996 PMC5444467

[B5] Bradl M Misu T Takahashi T Watanabe M Mader S Reindl M Adzemovic M Bauer J Berger T Fujihara K Itoyama Y Lassmann H Neuromyelitis optica: pathogenicity of patient immunoglobulin in vivo Ann Neurol 2009 66 630 643 10.1002/ana.21837 19937948

[B6] Kinoshita M Nakatsuji Y Kimura T Moriya M Takata K Okuno T Kumanogoh A Kajiyama K Yoshikawa H Sakoda S Neuromyelitis optica: Passive transfer to rats by human immunoglobulin Biochem Biophys Res Commun 2009 386 623 627 10.1016/j.bbrc.2009.06.085 19545538

[B7] Swanborg RH Experimental autoimmune encephalomyelitis in the rat: lessons in T-cell immunology and autoreactivity Immunol Rev 2001 184 129 135 10.1034/j.1600-065x.2001.1840112.x 12086308

[B8] Jarius S Wildemann B AQP4 antibodies in neuromyelitis optica: diagnostic and pathogenetic relevance Nat Rev Neurol 2010 6 383 392 10.1038/nrneurol.2010.72 20639914

[B9] Jones MV Nguyen TT Deboy CA Griffin JW Whartenby KA Kerr DA Calabresi PA Behavioral and pathological outcomes in MOG 35–55 experimental autoimmune encephalomyelitis J Neuroimmunol 2008 199 83 93 10.1016/j.jneuroim.2008.05.013 18582952

[B10] Hinson SR McKeon A Fryer JP Apiwattanakul M Lennon VA Pittock SJ Prediction of neuromyelitis optica attack severity by quantitation of complement-mediated injury to aquaporin-4-expressing cells Arch Neurol 2009 66 1164 1167 10.1001/archneurol.2009.188 19752309

[B11] Takahashi T Fujihara K Nakashima I Misu T Miyazawa I Nakamura M Watanabe S Shiga Y Kanaoka C Fujimori J Sato S Itoyama Y Anti-aquaporin-4 antibody is involved in the pathogenesis of NMO: a study on antibody titre Brain 2007 130 1235 1243 10.1093/brain/awm062 17449477

[B12] Cayrol R Saikali P Vincent T Effector functions of antiaquaporin-4 autoantibodies in neuromyelitis optica Ann N Y Acad Sci 2009 1173 478 486 10.1111/j.1749-6632.2009.04871.x 19758189

[B13] Frohman EM Kerr D Is neuromyelitis optica distinct from multiple sclerosis?: something for “lumpers” and “splitters” Arch Neurol 2007 64 903 905 10.1001/archneur.64.6.903 17562944

[B14] Weinstock-Guttman B Miller C Yeh E Stosic M Umhauer M Batra N Munschauer F Zivadinov R Ramanathan M Neuromyelitis optica immunoglobulins as a marker of disease activity and response to therapy in patients with neuromyelitis optica Mult Scler 2008 14 8 1061 1067 10.1177/1352458508092811 18573816

[B15] Kinoshita M Nakatsuji Y Kimura T Moriya M Takata K Okuno T Kumanogoh A Kajiyama K Yoshikawa H Sakoda S Anti-aquaporin-4 antibody induces astrocytic cytotoxicity in the absence of CNS antigen-specific T cells Biochem Biophys Res Commun 2010 394 205 210 10.1016/j.bbrc.2010.02.157 20188706

[B16] Lapointe BM Herx LM Gill V Metz LM Kubes P IVIg therapy in brain inflammation: etiology-dependent differential effects on leucocyte recruitment Brain 2004 127 2649 2656 10.1093/brain/awh297 15355874

[B17] Tani T Sakimura K Tsujita M Nakada T Tanaka M Nishizawa M Tanaka K Identification of binding sites for anti-aquaporin 4 antibodies in patients with neuromyelitis optica J Neuroimmunol 2009 211 110 113 10.1016/j.jneuroim.2009.04.001 19410301

